# Designing long lifespan aqueous Zn-ion battery by learning from natural ion channels

**DOI:** 10.1093/nsr/nwae260

**Published:** 2024-07-29

**Authors:** Huijun Zhao

**Affiliations:** Centre for Catalysis and Clean Energy, Gold Coast Campus, Griffith University, Australia

Aqueous Zn-ion batteries (AZIBs) have been regarded as an indispensable class of energy storage devices for sustainable energy systems because of their low cost, considerable storage capacity and rate capability, environmentally friendly nature, and importantly, high safety characteristics even when damaged or under extreme environmental conditions. Ascribed by the challenges of detrimental side reactions of dendrite growth, corrosion, and hydrogen evolution reaction (HER) occurring during service, for real world applications, AZIBs need to overcome many hurdles [1,2]. It is fortunate that the philosophy of learning from nature opens a new means to overcome these hurdles through designing and constructing advanced bioinspired materials and interfaces [3,4]. In this regard, the team led by Professor Ziqi Sun has made noticeable contributions [5,6].

**Figure 1.**
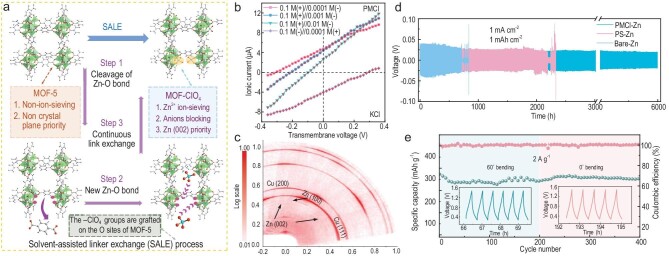
(a) Fabrication of bioinspired membrane via a solvent-assisted linker exchange (SALE) process; (b) *I–V* curves of bioinspired membrane measured at various KCl concentration gradients; (c) X-ray WR-RSM mapping confirming the Zn (002) texture; (d) cycling performance of symmetric cells at 1 mA cm^−2^ and 1 mAh cm^−2^; (e) pouch cell performances under bendings.

Inspired by the biological cell potassium channels (KcsA), very recently, Ting Liao, Lei Jiang, Ziqi Sun and their team proposed and demonstrated the use of a delicate design of ultrafluidic and selective artificial secondary electronic interphase (SEI) to realize dendrite-free and ultralong lifespan AZIBs [7]. In biological cells, there are enzyme-gated serving channels in the cell plasma membranes, such as the typical K^+^ serving nanosized channels, which allow directional ultrafast K^+^ flux with ultrahigh ion selectivity to repel other ions from the penetration. Inspired by this biological channel, a Zn-ion selective bioinspired membrane (PMCl) was designed by grafting strongly electronegative –ClO_4_ groups onto the nanochannels of a metal–organic framework structure (MOF-5) (Fig. 1a). Similar to the natural potassium KcsA channels, the bioinspired PMCl membrane shows excellent cation (K^+^) selectivity and anion (Cl^−^) screening ability, as indicated by the drift-diffusion *I-V* curves with various concentration differences (Fig. 1b). For an aqueous ZnSO_4_ electrolyte in AZIBs, the bioinspired membrane can readily achieve an ultrafluidic Zn^2+^ flux of 1.9 × 10^−3^ mmol m^−2^ s^−1^ and a perfect Zn^2+^/SO_4_^2−^ ion selectivity. Furthermore, the strong hydrophobic nature of the bioinspired membrane reduces the direct contact of water with the Zn anode, which retards the corrosion and hydrogen generation of the side reaction between the aqueous electrolyte and active Zn metal.

When the bioinspired membrane as an artificial SEI is assembled on the top of Zn anode, a regulated Zn deposition behavior contributed by the ultrafast Zn^+^ transport and selectivity is successfully realized, by which a horizontal (002) orientation-preferred non-dendritic Zn-plating onto the Zn anode is observed in both the symmetrical and asymmetric cells (Fig. 1c). As expected, contributing to the non-dendritic Zn deposition and the retarded corrosion and hydrogen generation of the Zn electrode, the coin cell delivers an ultralong lifespan up to 6000 h at 1 mA cm^−2^/1 mAh cm^−2^ (Fig. 1d), and the flexible pouch cell composed of a V_2_O_5_ cathode and a Zn anode displays undegradable discharge capacity under deformations (Fig. 1e) and maintains high stability over 2000 cycles.

In summary, this work showcases a tangible way to seek solutions from well-evolved mother nature towards critical challenges in emerging sustainable energy technologies and sheds light on driving the sustainable aqueous non-toxic metal batteries into real-world applications. This bioinspired design of the artificial SEI could also be a general approach that is applicable when designing other active metal-based rechargeable batteries.
